# Trends of blood pressure and heart rate in normal pregnancies: a systematic review and meta-analysis

**DOI:** 10.1186/s12916-019-1399-1

**Published:** 2019-09-11

**Authors:** Lise Loerup, Rebecca M. Pullon, Jacqueline Birks, Susannah Fleming, Lucy H. Mackillop, Stephen Gerry, Peter J. Watkinson

**Affiliations:** 1 Department of Engineering Science, Oxford Institute of Biomedical Engineering, University of Oxford, Old Road Campus Research Building, Oxford, OX3 7DQ UK; 2 Centre for Statistics in Medicine, University of Oxford, Botnar Research Centre, Windmill Road, Oxford, OX3 7LD UK; 3 Nuffield Department of Primary Care Health Sciences, Radcliffe Primary Care Building, Radcliffe Observatory Quarter, Woodstock Rd, Oxford, OX2 6GG UK; 4 Nuffield Department of Women’s and Reproductive Health, Oxford University Hospitals NHS Foundation Trust, Headley Way, Oxford, OX3 9DU UK; 5 Centre for Statistics in Medicine, University of Oxford, Botnar Research Centre, Windmill Road, Oxford, OX3 7LD UK; 6 Kadoorie Centre for Critical Care Research and Education, Nuffield Department of Clinical Neurosciences, NIHR Oxford Biomedical Research Centre, Oxford University Hospitals NHS Foundation Trust, Oxford, OX3 9DU UK

**Keywords:** Pregnancy (MeSH), Vital signs (MeSH), Blood pressure (MeSH), Heart rate (MeSH), Maternal physiology

## Abstract

**Background:**

Current reference ranges for blood pressure and heart rate throughout pregnancy have a poor evidence base.

**Methods:**

This is a systematic review and meta-analysis. We included studies measuring blood pressure or heart rate from healthy pregnant women within defined gestational periods of 16 weeks or less. We analysed systolic blood pressure, diastolic blood pressure and heart rate by gestational age. We assessed effects of measurement year and method.

**Results:**

We included 39 studies undertaken in 1967–2017, containing 124,349 systolic measurements from 36,239 women, 124,291 diastolic measurements from 36,181 women and 10,948 heart rate measurements from 8317 women. Mean (95% CI) systolic blood pressure was lowest at 10 weeks gestation, 110.4 (108.5, 112.3) mmHg, rising to 116.0 (113.6, 118.4) mmHg at 40 weeks, mean (95% CI) change 5.6 (4.0, 7.2) mmHg. Mean (95% CI) diastolic blood pressure was lowest at 21 weeks gestation, 65.9 (64.2, 67.7) mmHg; rising to 72.8 (71.0, 74.6) mmHg at 40 weeks, mean (95% CI) change 6.9 (6.2, 7.5) mmHg. Mean (95% CI) heart rate rose from 79.3 (75.5, 83.1) beats/min at 10 weeks to 86.9 (82.2, 91.6) beats/min at 40 weeks gestation, mean (95% CI) change 7.6 (1.8, 13.4) beats/min. Studies using manual measurement reported higher diastolic blood pressures than studies using automated measurement, mean (95 CI) difference 4.9 (0.8, 8.9) mmHg. Diastolic blood pressure increased by 0.26 (95% CI 0.10–0.43) mmHg/year. Including only higher-quality studies had little effect on findings, with heterogeneity remaining high (*I*^2^ statistic > 50%).

**Conclusions:**

Significant gestational blood pressure and heart rate changes occur that should be taken into account when assessing pregnant women. Commonly taught substantial decreases in blood pressure mid-pregnancy were not seen and heart rate increases were lower than previously thought. Manual and automated blood pressure measurement cannot be used interchangeably. Increases in diastolic blood pressure over the last half-century and differences between published studies show contemporary data are required to define current normal ranges.

**Study registration:**

PROSPERO CRD42014009673

**Electronic supplementary material:**

The online version of this article (10.1186/s12916-019-1399-1) contains supplementary material, which is available to authorized users.

## Background

Heart rate and blood pressure are key vital signs for the assessment of pregnant women [1, 2]. Our understanding of the normal thresholds for these vital signs underpins their use. Many modern clinical guidelines do not reference the sources of their normal vital sign ranges [3–5]. Others refer to an obstetric physiology textbook, which references data from small individual studies published between 1970 and the mid-1990s [6]. Core textbooks commonly suggest a mid-pregnancy dip of around 5 mmHg for systolic and 10–15 mmHg for diastolic blood pressure along with a progressive rise in heart rate ranging from 10 to 20 beats/min [7–9]. Where referenced, these texts rely on the same physiology textbook [6] or small individual studies from over 30 years ago [10]. The changes in vital signs that occur in pregnancy are known to complicate the recognition of deterioration [11]. However, we could not identify a clinical guideline or Modified Early Obstetric Warning Score (MEOWS) [2] that took account of expected gestational changes in vital sign physiology. MEOWS use the perceived normal thresholds to determine whether a woman requires review. However, the thresholds used are based on clinical consensus [4, 12]. Small changes in thresholds make substantial differences to the ability of clinical scores to identify physiological deterioration [13, 14]. Evidence-based normal values that take into account changes during pregnancy are therefore required. We carried out a systematic review and meta-analysis to establish whether gestation-specific normal ranges for heart rate and systolic and diastolic blood pressure can be produced from available studies of participants that were “healthy” at recruitment. We planned to investigate the effects of year of measurement, method of measurement (for blood pressure) and parity on vital signs.

## Methods

This systematic review follows the Meta-analysis Of Observational Studies in Epidemiology (MOOSE) [15] and Preferred Reporting Items for Systematic Reviews and Meta-Analyses (PRISMA) [16] guidelines (Additional file 1). We registered the review (PROSPERO: CRD42014009673) and published the protocol [17].

We included cross-sectional, case-control or longitudinal studies containing at least fifty participants and where measurements were taken by a healthcare professional.

We included studies containing blood pressure or heart rate measurements from pregnant women recruited as “healthy”. We defined “healthy” as women not known to have conditions likely to affect blood pressure or heart rate at the point of first measurement, according to inclusion and exclusion criteria described in Table 1. We included studies where participants recruited as healthy subsequently developed conditions potentially affecting blood pressure or heart rate. In studies where these participants’ data were presented separately, we extracted vital sign measurements for both groups. We excluded data where an intervention potentially affecting these vital signs was studied. We included baseline measurements prior to an intervention. We excluded data from subgroups selected at recruitment on the basis of characteristics or medical diagnoses (Additional file 2: List S1); we extracted data from control groups of “healthy” pregnant women in these studies. We excluded studies where the gestational age at which measurements were taken was not defined to 16 weeks or less.

**Table 1:** Inclusion and exclusion criteria

Inclusion criteria	Exclusion criteria
Cross-sectional, case-control or longitudinal study	Measurements from women with illnesses likely to affect the cardiac or respiratory systems^‡^
Minimum of 50 patients	Measurements from women who were recruited because they were considered to be at high-risk of developing a pregnancy complication
Age 14 years or older	Measurements from women known to be taking medication which could affect the measurements
Objective measurement^*^ of heart rate and/or blood pressure	Measurements from women where the reported gestational age at the point of measurement was not defined in terms of days or weeks of gestation
Measurements taken during the antenatal period, up to the start of the intrapartum period^†^	Measurements from women where the time window in which the measurement was taken was not defined to within 16 weeks
Raw data or average measure reported and possible to extract within minimum accuracy	Measurements from self-monitoring or other measurements not taken by a healthcare professional
	Measurements from women with less than 10% singleton pregnancies
	For women known to undergo fertility procedures, any measurements taken prior to a positive pregnancy test
	Any of the following measurements (without valid baseline): • Measurements taken using ambulatory technologies • Measurements taken using invasive technologies • Measurements taken during anaesthesia • Measurements taken during sleep • Measurements taken during exercise • Measurements taken at heights greater than 1000 m above sea level

^*^An overview of acceptable measurement techniques has been described previously [17]

^†^Defined as progressive cervical dilatation with regular contractions

^‡^List of characteristics or diagnoses leading to exclusion are shown in Additional file 2: List S1

We set out to include measurements of all six vital signs recommended for clinical assessment (systolic blood pressure, diastolic blood pressure, heart rate, oxygen saturation, respiratory rate and/or temperature), made antepartum, intrapartum or postpartum [17]. We have restricted this report to blood pressure and heart rate data measured in the antepartum period, as we found little data for other vital signs or time periods.

### Search strategy

With a trained librarian, we searched MEDLINE, Embase and CINAHL, from inception until February 2018. We also searched reference lists and contacted field experts. Where required data were not presented or were presented in a form we could not extract, we contacted the original authors by e-mail. We used both MeSH and free-text terms. We did not restrict the year or language of publication. The complete search strategy has been described previously [17]. Additional file 2: Table S1 details the MEDLINE search strategy.

Two reviewers (LL and RP) independently screened retrieved titles and abstracts to exclude studies that clearly fell outside the scope of the review, such as foetal or animal studies. Two reviewers (PW and LM) independently screened the remaining studies by title, then by abstract and finally by full text against the inclusion and exclusion criteria (Table 1). Where reviewers disagreed, the study proceeded to the next screening stage.

### Data extraction

Two reviewers (LL and RP) independently extracted study data onto a piloted spreadsheet (Microsoft Excel). Disagreements were automatically highlighted and resolved by recourse to the original papers or in consultation with a medical statistician (JB), intensive care specialist (PW) or obstetric physician (LM), as required.

For each study, we extracted information about the study (year of data collection and publication, study setting, country of study, data collection schedule), measurements (method, subject position, details of measurement device) and participants (age, weight, body mass index (BMI), ethnicity, reason for measurements, parity, number of gestations, pregnancy dating method). For each period of pregnancy defined in a paper, we extracted information about the sample size and minimum and maximum gestational age, together with reported summary statistics for blood pressure and heart rate. We used Engauge Digitizer, https://sourceforge.net/projects/digitizer/works (open source software) to extract data from graphs if the underlying data were not presented and we could not obtain it from the authors.

### Assessment of bias

Two reviewers (LL and RP) independently undertook quality assessment in line with QUADAS (Quality Assessment of Diagnostic Accuracy Studies)-2 [18], adapted from the methodology of Ioannou et al. [19] following pre-determined rules (Additional file 2: Table S2). We assessed studies over two domains: study design and reporting methods. We scored statistical methods within these two domains, taking account of differing study designs. Each methodological criterion was scored as either “high” or “low” risk of bias. For each of the two domains, the overall quality score for each study was defined as the percentage of “low risk of bias” marks over the total number of criteria. We assessed the effects of only including studies scoring 50% or more on mean estimates of heart rate and blood pressure and on heterogeneity between studies.

### Summary measures

Principal summary statistics were mean and standard deviation for each measure of heart rate and blood pressure, along with sample size. Where mean and standard deviation for blood pressure and heart rate were not reported, we approximated these assuming a normal distribution (with no skewness), in line with previous descriptions of vital sign measurement distributions [20]. Where multiple measurements were reported on the same day for the same participants (e.g. blood pressure measured in the sitting and lying positions), we selected a single data point using pre-specified rules [17]. We assigned the development status of a study country using the Human Development Index (2015) [21].

Where the gestational age associated with each data point was reported as a mean or median gestational age, we used this for the analysis. Where the gestational age associated with each data point was reported as a range, we used the mid-point of the range. Gestational age measured using ultrasound measurement of the foetal crown-rump length before 14 weeks gestation was our preferred assessment method [22].

Where the sample size associated with each data point was reported, we used this for the analysis. For studies where the sample size was reported only at baseline, we took the sample size associated with each data point to be the baseline sample size, assuming no dropouts or loss-to-follow-up.

Where characteristics of women (weight, BMI, age, parity) were reported at multiple time points (e.g. pre-pregnancy, at recruitment and/or at delivery), we used the value closest to recruitment. Where the mean values of the characteristics of the sample of women associated with each data point were reported, we used this for analysis. For studies where the characteristics of women were only reported for the entire sample of women recruited, and not for the sample at each time point, we used entire sample values, assuming no dropouts or loss to follow-up. Where only median values were reported, we used these instead of the mean values, assuming normal distributions of characteristics (with no skewness).

The study year was taken to be the last year of recruitment to the study or the year of publication where this was not reported.

### Synthesis of results

To analyse trajectories of changes in blood pressure and heart rate, we adapted the method for meta-analysis of longitudinal studies proposed by Ishak et al. [23, 24] Ishak conducted a meta-analysis of longitudinal studies reporting data at a series of fixed time points, using study summary statistics. Linear mixed effects models were used to take account of correlations in longitudinal data. We adapted Ishak’s general multivariate model to conduct a meta-analysis of studies which report data at different time points, whilst still accounting for correlations between data points within studies and between studies (our “longitudinal model”). The time points in our analysis are the different values of gestational age. All data points from an individual study were included. We used a random coefficients model that allows for an arithmetic description of the relationship between the measurement of interest and gestational age [25]. The models were built up based on polynomials of gestational age by adding higher order polynomials, first as fixed effects, and then as random coefficients. Decisions on which terms to include were based on AIC and BIC (Akaike and Bayesian Information Criterion)—measures of assessing model fit whilst accounting for the number of parameters [26, 27]. To assess the additional benefit of higher order fixed effects terms, we fitted models based on maximum likelihood (ML) estimation; whereas to assess the benefit of higher-order random effect terms, and to fit the final model, we undertook restricted maximum likelihood estimation (REML). Each observation was weighted according to the study reported standard error. To account for the correlation of observations within a study, a spatial power covariance structure was used, which allows for the irregular nature of time between measurements [28].

We undertook pre-specified secondary analyses to investigate the effects of method of measurement (manual vs. automated), year of data collection and parity on systolic and diastolic blood pressure. We added these covariates to the model as fixed effects. We added method of measurement and year of measurement simultaneously as we expected them to be related. To assess the effect of parity, where possible, we separated data into nulliparous and multiparous groups. Otherwise, we entered parity into the model as the proportion of multiparous mothers. We did not undertake secondary analyses of heart rate, as the limited study numbers would have resulted in imprecise results.

Additionally, we pooled data for each trimester from each included study in a random effects analysis to generate Forest plots for each vital sign. Trimesters were defined as trimester 1 from 0^+ 0^ to 12^+ 6^ weeks; trimester 2 from 13^+ 0^ to 25^+ 6^ weeks; trimester 3 from 26^+ 0^ to 36^+ 6^ weeks and full term from 37^+ 0^ weeks to delivery [29]. Where data were reported for subgroups within a single study, for example for nulliparous and multiparous women, a weighted mean and standard error of the mean were calculated. Where more than two data points for a single trimester within a study were reported, the same women would be included in both data points, so the mean of the data points was calculated. The standard error of the mean was calculated assuming a correlation between measurements on the same patient of 0.7. This was considered reasonable considering how close in time the measurements were. The weighted mean of the gestational age for the data points was calculated.

We estimated the pooled weighted mean and 95% confidence intervals where the weight of an individual study was inversely proportional to the sum of the variance (SE^2^) of the study mean and the between-study variance. To estimate the spread of vital sign data in an individual study setting, we computed 95% prediction intervals from the random effects analysis, according to the methodology presented by Riley et al. [30] We determined heterogeneity using the *I*^2^ statistic (range 0–100%).

We performed a pre-specified sensitivity analysis excluding outlying studies (with means lying outside of the predictive interval). We also performed an additional sensitivity analysis excluding studies scoring less than 50% on quality assessment.

As the studies included in the review did not have a comparative design, we could not assess publication bias. However, selective reporting was assessed as part of the quality assessment.

Statistical analyses were undertaken using StataCorp. 2015 (Stata Statistical Software: Release 14. College Station, TX: StataCorp LP) or SAS software (Version 9.3, SAS Institute Inc. SAS).

## Results

**Fig. 1:**
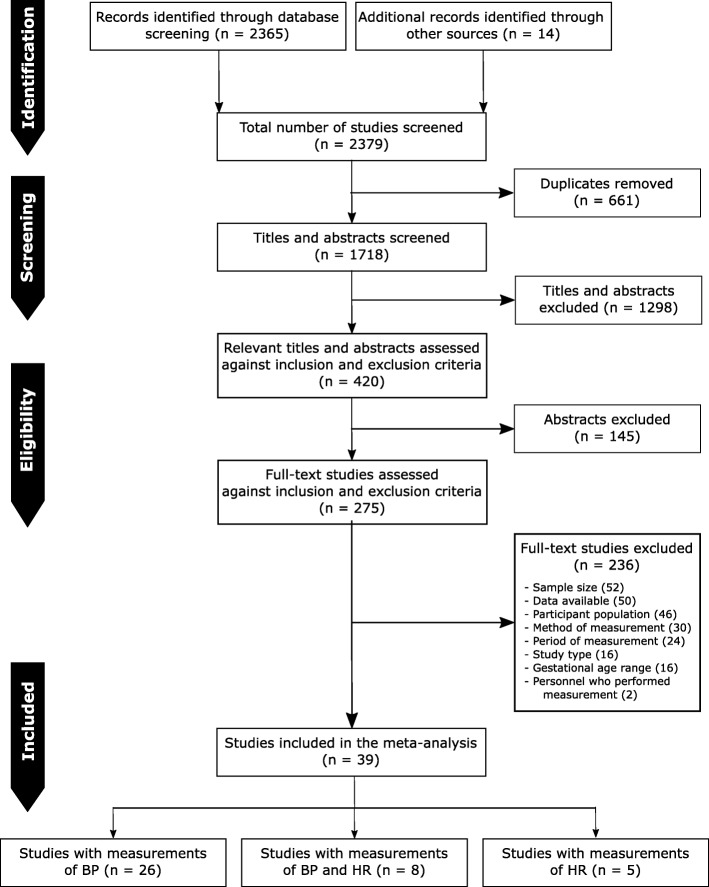
Study identification and selection

Database screening identified 1897 publications. We identified a further 14 publications from reference lists or expert knowledge. We included 39 studies from 20 countries meeting our pre-specified criteria, Fig. 1. Detailed reasons for exclusion are shown in Additional file 2: Table S3.

The included studies are summarised in Additional file 2: Table S4. We extracted blood pressure data from 34 studies. We extracted heart rate data from 13 studies. Studies came from 20 different countries, 17 high-development countries and three middle- or low-development countries. One (Russian) study was not reported in English [31].

We included 394 summary measurements of blood pressure, representing 124,349 systolic measurements from 36,239 women and 124,291 diastolic measurements from 36,181 women. The 23 longitudinal studies reported measurements of blood pressure from 33 separate groups of women [32–54]. Longitudinal studies varied in the number of time points at which a group had blood pressure measured (median 4 time points, range 2 to 35). A further 11 studies reported cross-sectional measurements [31, 55–64]. Two of these studies reported multiple cross-sectional measurements [56, 61]. The remaining studies reported measurements at a single time. A manual sphygmomanometer was used in fifteen studies [34, 35, 37–39, 41, 44, 45, 47, 49, 53, 57, 58, 62, 64]. An automated blood pressure monitor was used in thirteen studies [32, 33, 42, 43, 48, 50–52, 54–56, 59, 61]. Six studies did not report the measurement methodology [31, 36, 40, 46, 60, 63].

We included 34 summary measurements of heart rate, representing 10,948 heart rate measurements from 8317 women. The five longitudinal studies reported measurements from seven separate groups of women [32, 42, 48, 65, 66]. Summary measurements were reported at between 3 and 8 (median 4) time points per group. A further eight studies reported cross-sectional measurements of heart rate at a single time point [31, 57, 59, 63, 64, 67–69]. The method of heart rate measurement was reported as electrocardiography (ECG; 5 studies, 8 time points) [57, 64, 66–68], echocardiography (echo; 1 study, 2 time points) [69], finger arterial pressure sensor (1 study, 4 time points) [48], radioulnar pulse wave monitoring (1 study, 8 time points) [65], reading from an automated blood pressure (BP) monitor (2 studies, 4 time points) [42, 59] or not specified (3 studies, 16 time points) [31, 32, 63].

Quality assessment scores ranged from 5.6 to 84.2% (median 44.4%, where 100% is a good score). Additional file 2: Figure S1 summarises the score per assessment criterion. Individual paper scores are shown in Additional file 2: Table S4. Common reasons for scoring poorly included not sufficiently defining the population under study (28 studies), not defining gestational age using ultrasound measurement of crown-rump length before 14 weeks (26 studies) and not describing/using measurement devices ratified for use in pregnancy (30 studies). Of the 13 studies that used automated methods of blood pressure measurement, three stated that they used machines validated for use in pregnancy [42, 48, 59]. Five studies reported that the measurement device was calibrated prior to the study taking place (all manual sphygmomanometer methods) [37, 39, 44, 62, 64]. The method of blood pressure measurement was not specified in six studies [31, 35, 36, 40, 46, 69]. Use of an appropriately sized BP cuff was reported in 13 studies [33, 37, 38, 42–45, 47, 49, 54, 58, 61, 62].

**Fig. 2:**
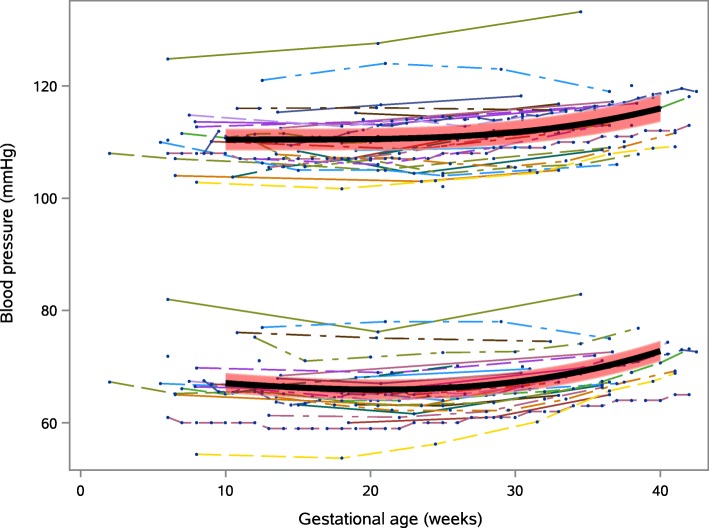
Mean BP (solid black line), with 95% CI (red band) by gestational age (longitudinal model). Trajectories of individual studies are also shown (thin lines)

**Fig. 3:**
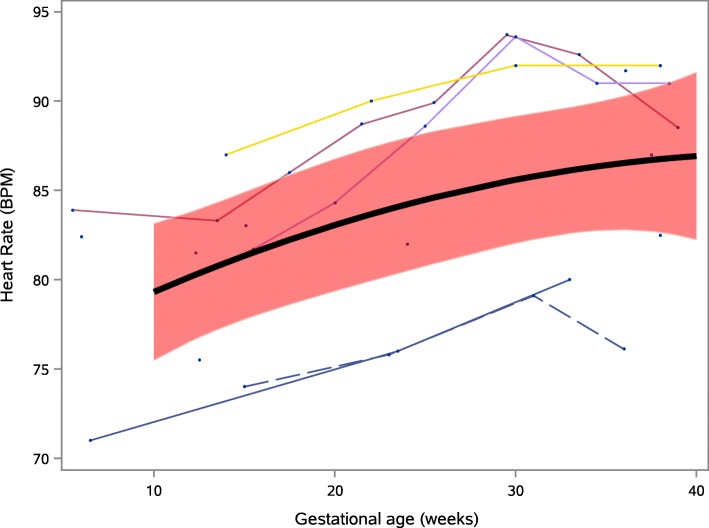
Mean heart rate (solid black line), with 95% CI (red band) by gestational age (longitudinal model). Trajectories of individual studies are also shown (thin lines)

Both systolic and diastolic blood pressure changed significantly across pregnancy (for combined manual and automatic readings) when modelled using our longitudinal model (Fig. 2). Systolic blood pressure was lowest at 10 weeks gestation, mean (95% confidence intervals, CI) 110.4 (108.5, 112.3) mmHg, rising to 116.0 (113.6, 118.4) mmHg at 40 weeks gestation; a change of 5.6 (95% CI 4.0, 7.2) mmHg from 10 to 40 weeks (*p* < 0.001). Mean (95% CI) diastolic blood pressure was 67.1 (65.4, 68.7) mmHg at 10 weeks gestation. Mean (95% CI) diastolic blood pressure was lowest at 21 weeks 65.9 (64.2, 67.7) mmHg, rising to 72.8 (71.0, 74.6) mmHg at 40 weeks; a change of 6.9 (95% CI 6.2, 7.5) mmHg from 21 to 40 weeks (*p* < 0.001). Mean (95% CI) heart rate rose from 79.3 (75.5, 83.1) beats/min at 10 weeks to 86.9 (82.2, 91.6) beats/min at 40 weeks; a change of 7.6 (1.8, 13.4) beats/min from 10 to 40 weeks (*p* = 0.014), Fig. 3. Though all longitudinal studies showed heart rate to increase across pregnancy, three relatively small studies showed a small drop in heart rate at the end of pregnancy [32, 48, 65].

Forest plots of systolic, diastolic and heart rate data (one plot per trimester) are shown (Additional file 2: Figure S2). Significant heterogeneity was present for all measures in all trimesters. Blood pressure and heart rate mean trimester estimates were similar whether estimated using our longitudinal model or random effects meta-analyses (Additional file 2: Table S5).

### Subgroup analyses

Studies that used a manual measurement reported higher mean diastolic blood pressures than studies using an automated technique, difference (95% CI) in mean intercept of 4.9 (0.8, 8.9) mmHg (*p* = 0.020). Mean manual systolic blood pressure measurements did not differ from those in studies that used an automated measurement, difference (95% CI) in mean intercept of 2.8 (− 2.3, 7.9), *p* = 0.274 (Additional file 2: Figure S3).

Studies of automated measures commenced in 2002 becoming more common over time. We therefore analysed the effect of time on blood pressure for manual measurements only. Each year from 1969 to 2017, diastolic blood pressure increased by 0.26 (95% CI 0.10–0.43) mmHg, *p* = 0.003. Systolic blood pressure rose by 0.12 (95% CI − 0.09, 0.33) mmHg per annum, but the rise was not statistically significant (*p* = 0.256). Differences were not affected by gestational age.

Data was available from studies to allow us to assess the effects of parity on systolic and diastolic blood pressure. Nulliparous and parous women did not differ in either systolic blood pressure, mean (95% CI) difference − 0.32 (− 7.7, 7.1) mmHg, *p* = 0.927, or diastolic blood pressure, mean (95% CI) difference 1.30 (− 5.2, 7.8) mmHg, *p* = 0.671, (Additional file 2: Figure S4).

We could not adjust for maternal characteristics, such as age and weight, as such data were often missing or reported using different summary measures at different gestational ages.

### Sensitivity analyses

Excluding outlying studies (with means lying outside of the predictive interval) reduced the number of studies by a maximum of five studies for studies of blood pressure. As all the outlying studies were “high outliers”, the mean estimates of systolic and diastolic blood pressure were decreased by about 1 mmHg, but the pattern across gestational ages remained the same (Additional file 2: Table S5b). This analysis also removed the one relatively small study where blood pressure appeared to decrease toward the end of pregnancy [49]. No studies of heart rate had means outlying the predictive interval.

Analysing only studies with a quality score above 50% reduced the number of studies for analysis from 34 to 12 for blood pressure and from 13 to 7 for heart rate. We found little change in mean estimates of blood pressure (though systolic blood pressure fell marginally in trimester 2), or heart rate, and measures of heterogeneity remained above 95% (Additional file 2: Table S5c).

## Discussion

Our study synthesises the available evidence to describe changes in blood pressure and heart rate during pregnancy. As far as we are aware, this has not previously been done. We show that systolic blood pressure rises by 5.6 (95% CI 4.0, 7.2) mmHg between 10 and 40 weeks gestation (with a possible slight drop of around 1 mmHg in trimester 2 when only higher-quality studies were included). Unlike systolic blood pressure, diastolic blood pressure is lowest at 21 weeks gestation, rising by 6.9 (CI 6.2, 7.5) mmHg by 40 weeks. Mean diastolic blood pressures were on average 4.9 (0.8, 8.9) mmHg lower when measured using automated techniques than when using manual methods. There is no difference in either systolic or diastolic blood pressure between nulliparous and parous women. Over 48 years, average diastolic blood pressures rose by 0.26 (95% CI 0.10–0.43) mmHg per year. Heart rate rises by 7.6 (95% CI 1.8–13.4) beats per minute through pregnancy.

Our synthesis brings together more blood pressure and heart rate data from more pregnant women than has previously been done. This increases our understanding of the trends that occur during pregnancy. It also shows the large between-study heterogeneity, emphasising the importance of not simply taking results from a single study. Consequently, our mean estimates were imprecise (95% CI width was around 5 mmHg for blood pressure and 9 beats per minute for heart rate). We did not meta-analyse other centile estimates for blood pressure and heart rate (for example 5th or 95th). As most studies did not report centiles of the distributions, approximations would need to be made, using assumptions about data distributions. The precision would likely have been lower than at the means, resulting in wide confidence intervals, with little clinical value. Such an analysis would have been imprecise and potentially misleading.

As with many meta-analyses, combining these data is complex. Included studies were of mixed methodological quality. We identified three aspects of particular concern: Firstly, a lack of accuracy in measuring and reporting of gestational age. Secondly, only a minority of studies reported using automated blood pressure measurement devices ratified for use in pregnancy. This means that we cannot ascertain whether the differences between blood pressures measured with manual sphygmomanometers and automatic methods would have been present if automatic methods ratified for use in pregnancy had been used. Thirdly, many studies insufficiently defined the population studied. These methodological weaknesses may have contributed to the range of possible mean blood pressures for any gestational age in our analyses. They may also partly account for the heterogeneity we found. Sensitivity analyses to account for quality and outlying studies had little effect in reducing heterogeneity. Further exploration of the sources of the heterogeneity was prevented by a lack of consistent reporting of the additional variables that would be required. The substantial between-study difference in blood pressure and heart rates at all gestational ages creates uncertainty around our mean values, but is less of a problem when analysing trends across pregnancy and time and when considering differences between measurement methods.

We investigated the effect of methodological quality in two ways, by undertaking a pre-specified analysis excluding outlying studies and by excluding studies scoring less than 50% on quality assessment. As all the outlying studies were “high outliers”, the mean estimates of systolic and diastolic blood pressure were decreased by about 1 mmHg, but the pattern across gestational ages remained the same (Additional file 2: Table S5b). Excluding studies with a quality assessment score of less than 50% had little change in mean estimates of blood pressure (though systolic blood pressure fell marginally in trimester 2), or heart rate (Additional file 2: Table S5c).

We planned to estimate blood pressure and heart rate trends for normal pregnancies, accepting that definitions of normal pregnancy vary. Where participants subsequently developed complications, we included their readings. As a consequence, we kept in women who developed pre-eclampsia or gestational hypertension whilst in the original study. As most studies did not present data excluding these participants, we could not derive mean values for populations who remained “normal” at delivery.

We combined longitudinal and cross-sectional study data. Blood pressures from longitudinal studies are not independent, in contrast to cross-sectional studies where blood pressures from different women at different points in pregnancy are independent. To avoid underestimating the precision of our estimates by ignoring the longitudinal correlations, we used the random time-effects method proposed by Ishak et al. for the meta-analysis of summary data. This model addresses the dependencies between longitudinal data points through the inclusion of random time effects between studies. Additionally, the random time-effects method accounts for the exact measurement time points of the included studies. We could not include within-study correlations as none of the included studies reported these. Maternal age and weight were often not presented, and where presented, the gestational age at which they were reported was inconsistent, preventing exploration of their effect on the temporal increase in diastolic blood pressure. The included studies came from a wide range of countries (Additional file 2: Table S4), including a wide range of ethnicities. However, our results may not be generalisable across all ethnic groups. Given the large number of included patients, it appears unlikely that our pre-specified plan to exclude studies with less than 50 participants significantly affected our findings.

Our findings remain compatible with the known increase in myocardial alpha receptors (resulting in increased heart rate), increased plasma volume and decreased systemic vascular resistance that occur in pregnancy [9]. However, in comparison to the 5-mmHg mid-pregnancy drop for systolic blood pressure and 10–15-mmHg drop for diastolic blood pressure previously suggested [7–9], the changes we found were small (with little change in systolic blood pressure and a 2–3-mmHg drop in diastolic blood pressure). It is possible that blood pressure drops in pregnancy before most of the included studies took measurements [42, 70]. However, our lowest mean systolic and diastolic blood pressures were similar to those reported for normal young, non-pregnant female populations [71] and a study used as evidence of an early drop is included in our model [42]. Heart rate rose by 7.6 (95% CI 1.8, 13.4) beats/min rather than the 10–20 beats/min core texts commonly suggest [7–9]. Taken with other work showing smaller changes than previously though [38, 43, 51], these findings should alter current teaching, allowing more reliable assessment of pregnant women. Previous work in adult early warning scores shows that relatively small changes in thresholds have large changes in the ability of a score to detect deterioration [13, 14]. Published maternal early warning scores commonly have ranges for alerting thresholds of 10 mmHg for blood pressure and 10 beats per minute for heart rate [11, 72]. Our study shows that the normal changes in pregnancy account for over half these ranges. It seems likely that detection of acute deterioration in pregnancy could be improved by taking account of gestation. Equally, recognition of women at risk of hypertensive disorders of pregnancy may be improved by taking account of these patterns [73]. Prior to our study, possibly the best evidence for blood pressure patterns in pregnancy came from a single-centre study undertaken over 20 years ago [38]. Data from this work contributes to our study. The evidence for patterns in heart rate was even more limited. Blood pressure differences between nulliparous and parous women were small, in line with previous findings [38], and did not reach statistical significance.

Our finding of increasing diastolic blood pressure over the 48 years in which these studies have been undertaken is previously unknown. We did not find a study with data over a sufficient period to add to this finding. Increasing average maternal age [74–76] and/or weight [38, 77] may provide part of the explanation. These changes contribute to the uncertainty of our overall estimates.

The mean diastolic blood pressure was lower when measured using automated methods than when measured manually. Previously, several automated monitors have been found to underestimate diastolic blood pressure in pre-eclampsia [78–80]. Although we included women who subsequently developed pre-eclampsia, their contribution to our overall effect will be small as they form a small proportion of the total cohort and contributed readings prior to developing pre-eclampsia. Importantly, the mean difference is just lower than the minimum validation requirement for automated devices, suggesting that many of the devices used would not achieve this criteria [81]. The difference between manual and automated measurement could commonly change the weight applied to a blood pressure measurement in early warning scores. As many of the automated techniques were not known to be certified for use in pregnancy and several automated devices have been validated for use in pregnancy [82–85], it may be that the problem could be resolved by only using certified devices. Alternatively, the difference may in part be explained by use of the fourth rather than fifth phase to determine diastolic pressure manually.

Future studies should define gestational age precisely, recruit well-defined populations and for blood pressure only use devices ratified for use in pregnancy. For blood pressure, the differences between automated and manual methods across pregnancy need exploring with automated methods approved for use in pregnancy. The changes in diastolic blood pressure across five decades suggest normal ranges should be regularly revisited. Finally, the impact of using gestation-specific centiles to allow earlier detection of the unwell mother requires investigation.

## Conclusions

Gestational changes in blood pressure and heart rate should be taken into account when assessing pregnant women, but heterogeneity between studies prevents the production of gestation-specific evidence-based normal ranges. Assessment of blood pressure need not differ between nulliparous and parous women. Automatically measured diastolic blood pressures are lower than those measured manually. A consistent measurement system should be used through pregnancy. Decreases in blood pressure mid-pregnancy and increases in heart rate through pregnancy are smaller than previously thought. Taken with other work, these findings should contribute to more reliable assessment of pregnant women. Increases in diastolic blood pressures recorded over the last half century show contemporary data is required to define normal ranges for current practice.

## Additional files

Additional file 1: PRISMA checklist. (DOCX 30 kb)

Additional file 2: Exclusion diagnoses and reasons, search strategy, quality assessment criteria and results, summary of included studies, additional analyses. (DOCX 512 kb)

## Data Availability

All data generated or analysed during this study are included in this published article (and its supplementary information files).

## Abbreviations

AIC: Akaike Information Criterion
BIC: Bayesian Information Criterion
BMI: Body mass index
BP: Blood pressure
CI: Confidence interval
ECG: Electrocardiography
MEOWS: Modified Early Obstetric Warning Score
ML: Maximum likelihood
MOOSE: Meta-analysis Of Observational Studies in Epidemiology
PRISMA: Preferred Reporting Items for Systematic Reviews and Meta-Analyses
QUADAS-2: Quality Assessment of Diagnostic Accuracy Studies 2
REML: Restricted maximum likelihood estimation
