# Supplementary material for: Trends of blood pressure and heart rate in normal pregnancies: a systematic review and meta-analysis
Source: BMC Med. 2019 Sep 11;17:167. doi: 10.1186/s12916-019-1399-1 (PMC6737610; doi:10.1186/s12916-019-1399-1)
Supplement: Supplementary file 2 — Exclusion diagnoses and reasons, search strategy, quality assessment criteria and results, summary of included studies, additional analyses. (DOCX 512 kb) [file 12916_2019_1399_MOESM2_ESM.docx]

**Additional file 2: Exclusion diagnoses and reasons, search strategy, quality assessment criteria and results, summary of included studies, additional analyses**

**List SI**

Characteristics of medical diagnoses leading to exclusion:

- Known hypertension (chronic hypertension or recruitment after the development of gestational hypertension or pre-eclampsia)
- Diabetes mellitus, gestational diabetes

**Table S1**– Search strategy

| **Population** | **Physiological variables** | **Measurement** |
| --- | --- | --- |
| pregnan*.ti;  maternal.ti;  obstetric*.ti;  "expectant mother*".ti; "expecting mother*".ti; peripartum.ti;  "peri partum".ti; antepartum.ti;  "ante partum".ti; postpartum.ti;  "post partum".ti; intrapartum.ti;  "intra partum".ti; puerperium.ti;  trimester*.ti;  perinatal*.ti;  antenatal*.ti;  postnatal*.ti | "vital sign*".ti;  "early warning".ti;  EWS*.ti;  *"modified early obstetric warning".ti;*  *score*.ti;*  *MEOWS.ti;*  *chart*.ti*  physiolog*.ti; haemodynam*.ti; hemodynam*.ti; normogram*.ti;  "heart rate*".ti;  "pulse rate*".ti;  "pulse oximetry".ti;  "oxygen saturation*".ti; SpO2.ti;  Sp02.ti;  "blood pressure*".ti; (temperature AND body).ti; "respirat* rate*".ti;  *"breath* rate*".ti;*  *temperature*.ti;*  *breath*.ti;*  *respirat*.ti;*  *"cardiac rate*".ti;*  *oximetry.ti;* | trend*.ti;  pattern*.ti;  range*.ti;  change*.ti;  measur*.ti;  monitor*.ti;  record*.ti;  assess*.ti;  evaluat*.ti;  observ*.ti;  guidance.ti;  guideline*.ti;  technique*.ti;  method*.ti;  systematic*.ti;  Chart* |

The search strategy was designed with assistance of a qualified librarian in the Oxford University Health Care Libraries, following an initial search by two reviewers (LL and RP), review of the findings of the initial search with clinicians (PW and LM) and adjustment to improve detection of papers known to us.

### Limits applied to search:

- Human subjects
- Databases: Medline, Embase, CINAHL
- Publication date: Database inception to November 2016
- Abstract available in English

**Table S2**– Quality assessment criteria

|  | **Assessment criteria** | **Low risk of bias** | **High risk of bias** |
| --- | --- | --- | --- |
|  | **Study design criteria** | | |
| **1** | Study design | Clearly described as either cross-sectional or longitudinal/serial (accept follow-up, serial etc) | Not reported **OR** mixture of cross-sectional and longitudinal data |
| **2** | Sample selection | Population-based study where there are attempts to identify and clearly define populations from a specific geographical area **OR** women selected either consecutively or at random | Not population based **OR** convenience sampling **OR** arbitrary recruitment |
| **3** | Number of assessments (cross-sectional only) | Each woman was measured and included only once | Some women were measured and included more than once – would imply mixture of cross-sectional and longitudinal data |
| **4** | Gestational age interval of assessments (longitudinal only) | Interval of measures prospectively pre-specified and justified | Interval of measures not prospectively pre-specified and justified |
| **5** | Number of assessments (longitudinal only) | Clear documentation of the intended number of serial measurements | No clear documentation of the intended number of serial measurements |
| **6** | Inclusion/exclusion criteria | Women at high risk of pregnancy complications were not included **AND** all women were included in the final analysis | The study population included both low-risk and high-risk pregnancies **OR** women with abnormal outcome were excluded **OR** criteria not well explained |
| **7** | Sample size calculation | A priori determination of sample size and justification | Lack of a priori sample size determination and justification |
| **8** | Data collection time point | Prospective study | Retrospective study |
| **9** | Data collection purpose | Vital sign data collected (and analysed) specifically in order to investigate values or trends of vital sign in pregnancy | Data collected for another purpose (e.g. for validation of measurement equipment) |
| **10** | Pregnancy dating method | Clearly described **AND** a sonogram before 14 weeks | Sonogram after 14 weeks **OR** sonogram at unspecified gestational age **OR** sonogram not primary dating method |
| **11** | Collection of data on gestational age | The gestational age was calculated precisely to the day | Truncation of gestational age to the number of ‘completed weeks’ |
| **12** | Number of measurements per assessment | More than one assessment per woman per appointment | Single measure **OR** not specified |
| **13** | Suitability of equipment used | Any BP monitor used approved for pregnancy **OR** calibrated sphygmomanometer | No evidence of calibration of sphygmomanometer **OR** BP monitor not approved for pregnancy |
|  | **Reporting methods** | | |
| **14** | Characteristics of study population | Presented in a table or clearly described **AND** includes minimum dataset of age **AND** weight **AND** (height **OR** BMI) and parity | Not presented in a table **OR** not clearly described **OR** does not contain minimum dataset |
| **15** | Number of women approached and enrolled (prospective studies only) | Both described | Not described **OR** only number enrolled described |
| **16** | Details of equipment used | Manufacturer and model for each device stated **OR** mercury sphygmomanometer (in case of BP measurement) | Missing information about some or all devices |
| **17** | Qualification of observers | Measurement taken by healthcare professional **OR** trained personnel | Taken by anyone else **OR** unreported |
| **18** | Number of observers | Reported | Unreported |
| **19** | Description of measurement technique | The study described sufficient and unambiguous details of the measurement techniques used for vital sign recording; including position of subject. For the measurement of BP, a sitting position was specified **AND** the appropriate size of BP cuff was used. | Description insufficient **OR** ambiguous |
| **20** | Description of raw data | (Presented in a table **OR** clearly described) **AND** (Presented per week of gestation **OR** for a clearly defined period of gestation) | Not presented in a table **OR** not clearly / partly described |

**Table S3** – Reasons for exclusion of articles

| **Reason for rejection of studies** | **Relevant Inclusion/Exclusion criteria** | **Studies (n)** |
| --- | --- | --- |
| 1. Study type | Required to be a cross-sectional, case-control or longitudinal study | 16 |
| 2. Participant population | Exclude measurements from women with illnesses likely to affect the cardiac or respiratory systems* | 25 |
|  | Exclude women recruited as at high-risk of developing a pregnancy complication | 6 |
|  | Exclude measurements from women known to be taking medication which could affect the measurement | 0 |
|  | Exclude measurements from women with less than 10% singleton pregnancies | 2 |
|  | Studies must report a unique population | 13 |
| 3. Period of measurement | Measurements are required to be taken during the antenatal period, up to the start of the intrapartum period | 24 |
| 4. Method of measurement | Exclude measurements taken using ambulatory technologies or invasive technologies; during anesthesia, sleep or exercise; or at heights greater than 1000m above sea level, where there is no baseline measurement. | 24 |
|  | Measurement method and participant position must be acceptable (refer to Table 2 of protocol) | 6 |
| 5. Personnel who performed measurement | Exclude measurements from self-monitoring or other measurements not taken by a healthcare professional | 2 |
| 6. Data available | Studies must report an objective measurement of heart rate and/or blood pressure (systolic or diastolic blood pressure, mean arterial pressure is not acceptable). | 46 |
|  | Measurements must be able to be extracted within minimum accuracy (refer to Table 2 of protocol). | 5 |
| 7. Sample size | Minimum of 50 participants | 52 |
| 8. Gestational age specification | Exclude measurements from women where the reported gestational age at the point of measurement is not defined in terms of days or weeks of gestation. | 11 |
|  | Exclude measurements from women where the time window in which the measurement was taken was not defined to within 16 weeks. | 5 |
| **Total number of studies rejected** |  | **236** |

*Refer to Additional file 2: List S1

Note: Studies were assessed in order of criteria from 1 to 8. Therefore where studies could have been excluded for multiple reasons, they were classed in the first relevant category.

**Table S4** – Summary of studies included in systematic review

| **Reference** | **Year** | **Country ^a^** | **Design** | **Data collection** | **BP measurement** | **HR measurement** | **Measurement position** | **Total women (n)** | **Groups (n)** | **Time points per group (n)** | **Quality score (%)** |
| --- | --- | --- | --- | --- | --- | --- | --- | --- | --- | --- | --- |
| Amoakoh-Coleman *et al* | 2017 | Ghana | CS | P | automated | - | sitting | 361 | 1 | 1 | 55.6 |
| Andreas *et al.* | 2016 | Austria | LT | P | automated | other | lateral | 157 | 2 | 7 | 31.6 |
| Bakker *et al.* | 2010 | The Netherlands | LT | P | automated ^d^ | - | sitting | 7,106 | 3 | 3 | 57.9 |
| Carpenter *et al.* | 2015 | UK | CS | P ^b^ | - | ECG | supine | 99 | 1 | 1 | 52.9 |
| Churchill *et al.* | 1996 | UK | LT | P | manual ^c,d^ | - | sitting | 209 | 1 | 3 | 78.9 |
| Demidov *et al.* | 1978 | Russia | CS | P | nr | nr | lateral | 50 | 1 | 1 | 22.2 |
| Ekholm *et al.* | 1994 | Finland | CS | P | manual | ECG | sitting (BP); supine (HR) | 94 | 2 | 1 | 33.3 |
| Elvan-Taspinar *et al.* | 2003 | The Netherlands | CS | P | manual ^d^ | - | recumbent | 54 | 1 | 1 | 55.6 |
| Foo *et al.* | 2017 | UK | CS ^e^ | P | automated ^c^ | automated | sitting | 140 | 1 | 1 | 66.7 |
| Grindheim *et al.* | 2012 | Norway | LT | P | automated ^c^ | other | lateral | 57 | 1 | 4 | 57.9 |
| Gu *et al.* | 2006 | China | LT | R ^b^ | - | other | lateral | 182 | 2 | 8 | 41.2 |
| Guy *et al.* | 2018 | UK | CS | P | - | ECG | sitting | 2,586 | 1 | 1 | 61.1 |
| Halligan *et al.* | 1993 | Ireland | LT | P | manual ^d^ | - | sitting | 98 | 1 | 4 | 63.2 |
| Hilmert *et al.* | 2008 | USA | LT | P | automated | - | sitting | 164 | 1 | 3 | 47.4 |
| Ishikuro *et al.* | 2013 | Japan | LT | P ^b^ | automated | - | sitting | 587 | 3 | 8 | 42.1 |
| Iwasaki *et al.* | 2002 | Japan | LT | R ^b^ | automated | - | sitting | 1,570 | 1 | 3 | 16.7 |
| Jasovic-Siveska *et al.* | 2011 | Macedonia | LT ^b^ | P | manual | - | nr | 400 | 3 | 4 | 15.8 |
| Kac *et al.* | 2016 | Brazil | LT | P | automated ^d^ | - | sitting | 225 | 1 | 3 | 47.4 |
| Khan *et al.* | 2013 | India | LT ^b^ | P | manual | - | sitting | 200 | 3 | 3 | 26.3 |
| Lauszus *et al.* | 2007 | Denmark | LT ^b^ | P | manual | - | nr | 59 | 1 | 2 | 31.6 |
| Lee *et al.* | 2012 | USA | LT | P | nr | - | sitting | 1,684 | 1 | 2 | 21.1 |
| Lo *et al.* | 2002 | New Zealand | LT | P ^b^ | manual ^c,d^ | - | sitting | 101 | 1 | 4 | 52.6 |
| Macdonald-Wallis *et al.* | 2015 | UK | LT | R ^b^ | manual ^d^ | - | sitting | 13,016 | 1 | 18 | 27.8 |
| MacGillivray *et al*. | 1969 | UK | LT ^b^ | P ^b^ | manual ^c^ | - | sitting | 226 | 1 | 7 | 31.6 |
| Mahendru *et al.* | 2014 | UK | LT | P | automated ^c,d^ | automated | lateral | 54 | 1 | 3 | 84.2 |
| Matkin *et al.* | 1999 | USA | LT ^b^ | P | nr | - | nr | 2,853 | 2 | 35 | 10.5 |
| Miller *et al.* | 2007 | USA | LT | P | manual | - | sitting | 1,733 | 1 | 3 | 31.6 |
| Nama *et al.* | 2011 | UK | LT | P | automated | - | Sitting | 255 | 1 | 4 | 47.4 |
| Ngan *et al.* | 2007 | China | CS | P | nr | - | lateral | 58 | 1 | 1 | 38.9 |
| Ochsenbein-Kolble *et al.* | 2004 | Switzerland | CS | P | automated ^d^ | - | sitting | 3,234 | 37 | 1 | 66.7 |
| Okonofua *et al.* | 1992 | Nigeria | LT | P ^b^ | manual ^d^ | - | sitting | 189 | 1 | 6 | 36.8 |
| Peterson *et al.* | 1992 | USA | LT ^b^ | P ^b^ | nr | - | nr | 356 | 1 | 6 | 10.5 |
| Strevens *et al.* | 2001 | Sweden | LT | R ^b^ | manual ^d^ | - | sitting | 600 | 1 | 10 | 44.4 |
| Tape *et al.* | 1994 | USA | CS | P ^b^ | manual ^c,d^ | - | sitting | 59 | 1 | 1 | 61.1 |
| Tuffnell *et al.* | 1990 | UK | CS ^b^ | P ^b^ | nr | nr | sitting | 50 | 1 | 1 | 5.6 |
| Turan *et al.* | 2008 | UK | CS | P ^b^ | - | echo | lateral | 4,689 | 2 | 1 | 76.5 |
| van Der Graaf *et al.* | 2013 | Australia | CS | P | manual ^c^ | ECG | nr | 225 | 2 | 1 | 83.3 |
| van Oppen *et al.* | 1996 | The Netherlands | LT | P ^b^ | - | ECG | sitting | 50 | 1 | 4 | 50.0 |
| Veerabhadrappa et al. | 2015 | India | CS | P | automated | - | nr | 124 | 3 | 1 | 27.8 |
| Design: LT = longitudinal; CS = cross-sectional. Data collection: P = prospective; R = retrospective  Measurement type and position: nr = desired information not reported in paper  ^a^ Country of study (if stated), otherwise country of study group  ^b^ Study design (quality criterion 1) or data collection scheme (quality criterion 8) not explicitly stated or description unclear  ^c^ Measurement device suitable for use in pregnancy (quality criterion 13) either calibrated mercury sphygmomanometer or automated blood pressure monitor approved for use in pregnancy  ^d^ Appropriate BP cuff-size used (quality criterion 19)  ^e^ Although designed as a longitudinal study, only cross-sectional data available for inclusion | | | | | | | | | | | |

**Table S5** – Pooled estimates

Pooled estimates of systolic blood pressure, diastolic blood pressure and heart rate obtained from random effects meta-analyses (as displayed in Forest plots) and the modified Ishak model are summarised in the tables below. Table 5(a) shows the results when all studies are included. Tables 5(b) and 5(c) and the results of sensitivity analyses with outliers removed (b) and poor quality studies removed (c).

The mean estimate and lower and upper bounds of the 95% confidence interval are presented. *n* is the number of studies included for each category.

Table 5(a) – All studies

|  | | **Modified Ishak** | | | | **Random effects** | | | |
| --- | --- | --- | --- | --- | --- | --- | --- | --- | --- |
|  |  | ***n*** | **Mean** | **Lower** | **Upper** | ***n*** | **Mean** | **Lower** | **Upper** |
| *Systolic blood pressure (mmHg)* | Trimester 1 | 34 | 110.3 | 108.4 | 112.3 | 21 | 110.9 | 109.0 | 112.8 |
|  | Trimester 2 |  | 110.6 | 108.6 | 112.5 | 29 | 110.4 | 108.7 | 112.0 |
|  | Trimester 3 |  | 112.2 | 110.0 | 114.3 | 26 | 112.7 | 110.9 | 114.4 |
|  | Term |  | 116.0 | 113.6 | 118.4 | 12 | 113.0 | 110.8 | 115.2 |
| *Diastolic blood pressure (mmHg)* | Trimester 1 | 33 | 67.5 | 65.7 | 69.4 | 21 | 67.9 | 65.7 | 70.0 |
|  | Trimester 2 |  | 65.9 | 64.2 | 67.7 | 29 | 65.9 | 64.7 | 67.2 |
|  | Trimester 3 |  | 67.8 | 66.0 | 69.6 | 26 | 68.6 | 66.8 | 70.4 |
|  | Term |  | 72.8 | 71.0 | 74.6 | 11 | 70.2 | 68.6 | 71.8 |
| *Heart rate (bpm)* | Trimester 1 | 13 | 78.2 | 73.8 | 82.5 | 6 | 79.1 | 75.5 | 82.7 |
|  | Trimester 2 |  | 83.2 | 79.5 | 86.9 | 7 | 82.3 | 78.9 | 85.7 |
|  | Trimester 3 |  | 85.9 | 82.3 | 89.4 | 6 | 87.9 | 83.8 | 92.0 |
|  | Term |  | 86.9 | 82.2 | 91.6 | 4 | 87.2 | 84.1 | 90.4 |

Table 5(b) – All studies with outliers removed

|  | | **Modified Ishak** | | | | **Random effects** | | | |
| --- | --- | --- | --- | --- | --- | --- | --- | --- | --- |
|  |  | ***n*** | **Mean** | **Lower** | **Upper** | ***n*** | **Mean** | **Lower** | **Upper** |
| *Systolic blood pressure (mmHg)* | Trimester 1 | 34 | 109.6 | 108.0 | 111.1 | 19 | 109.6 | 107.9 | 111.2 |
|  | Trimester 2 |  | 109.7 | 108.1 | 111.2 | 27 | 109.3 | 107.7 | 110.8 |
|  | Trimester 3 |  | 111.2 | 109.4 | 113.0 | 23 | 111.7 | 110.1 | 113.4 |
|  | Term |  | 115.1 | 113.0 | 117.2 | 12 | 113.0 | 110.8 | 115.2 |
| *Diastolic blood pressure (mmHg)* | Trimester 1 | 31 | 66.5 | 65.0 | 67.9 | 19 | 67.8 | 66.2 | 69.4 |
|  | Trimester 2 |  | 65.0 | 63.6 | 66.4 | 24 | 65.4 | 64.5 | 66.3 |
|  | Trimester 3 |  | 66.8 | 65.4 | 68.2 | 25 | 68.0 | 66.8 | 69.2 |
|  | Term |  | 71.9 | 70.4 | 73.4 | 11 | 70.2 | 68.6 | 71.8 |
| *Heart rate (bpm)* | Trimester 1 | 13 | 78.2 | 73.8 | 82.5 | 5 | 78.5 | 74.6 | 82.3 |
|  | Trimester 2 |  | 83.2 | 79.5 | 86.9 | 7 | 82.3 | 78.9 | 85.7 |
|  | Trimester 3 |  | 85.9 | 82.3 | 89.4 | 5 | 87.1 | 80.8 | 93.4 |
|  | Term |  | 86.9 | 82.2 | 91.6 | 4 | 87.2 | 84.1 | 90.4 |

* Note for heart rate there were no outliers, so these values are the same as in Table 5(a)

Table 5(c) – High quality studies only (Quality score > 50%)

|  | | **Modified Ishak** | | | | **Random effects** | | | |
| --- | --- | --- | --- | --- | --- | --- | --- | --- | --- |
|  |  | ***n*** | **Mean** | **Lower** | **Upper** | ***n*** | **Mean** | **Lower** | **Upper** |
| *Systolic blood pressure (mmHg)* | Trimester 1 | 12 | 111.6 | 108.1 | 115.1 | 6 | 111.0 | 106.6 | 115.4 |
|  | Trimester 2 |  | 110.5 | 107.0 | 114.0 | 9 | 110.5 | 106.5 | 114.4 |
|  | Trimester 3 |  | 112.8 | 109.2 | 116.3 | 8 | 112.5 | 108.7 | 116.4 |
|  | Term |  | 115.4 | 111.8 | 118.9 | 1 | 111.6 | 110.9 | 112.3 |
| *Diastolic blood pressure (mmHg)* | Trimester 1 | 12 | 68.2 | 64.8 | 71.5 | 6 | 67.8 | 66.2 | 69.4 |
|  | Trimester 2 |  | 65.5 | 62.2 | 68.8 | 9 | 65.3 | 62.1 | 68.6 |
|  | Trimester 3 |  | 67.7 | 64.4 | 71.0 | 8 | 67.9 | 65.0 | 70.8 |
|  | Term |  | 72.6 | 69.2 | 76.0 | 1 | 64.9 | 64.4 | 65.4 |
| *Heart rate (bpm)* | Trimester 1 | 7 | 78.3 | 72.3 | 84.3 | 3 | 77.6 | 73.4 | 81.8 |
|  | Trimester 2 |  | 82.8 | 77.1 | 88.6 | 3 | 78.0 | 72.6 | 83.4 |
|  | Trimester 3 |  | 86.6 | 81.1 | 92.1 | 2 | 85.9 | 74.5 | 97.4 |
|  | Term |  | 89.4 | 83.0 | 95.8 | 0 | - | - | - |

**Figure S1** – Quality assessment scores per criterion


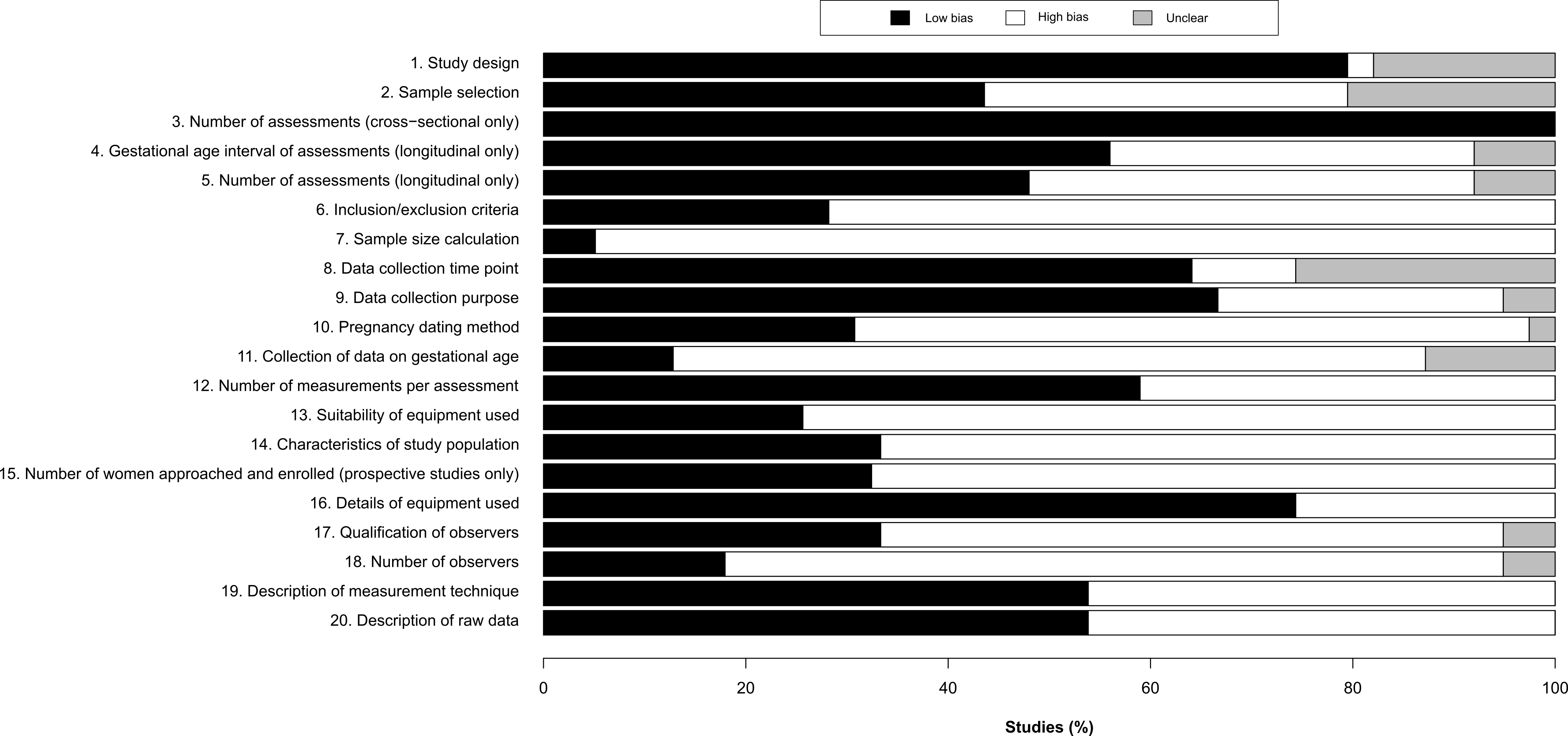


**Figure S2** – Forest plots of systolic, diastolic and heart rate data

Figure 2(a) – Systolic blood pressure (mmHg)

Figure S2(b) – Diastolic blood pressure (mmHg)

Figure S2(c) – Heart rate (bpm)

**Figure S3** – Subgroup analysis: by method of BP measurement. Trajectories of individual studies are also shown.

**Figure S4** – Subgroup analysis: by parity. Trajectories of individual studies are also shown
